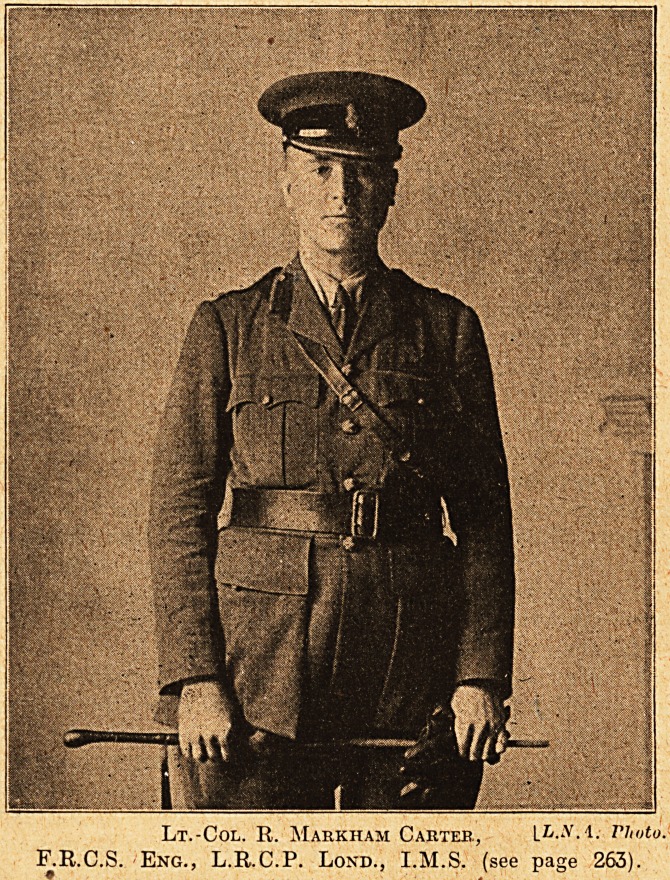# Hospital and Institutional News

**Published:** 1917-07-07

**Authors:** 


### HOSPITAL AND INSTITUTIONAL NEWS.

### THE OPENING OF BABY WEEK.

The "Baby Week" Campaign commenced on Monday, July 2, with the opening by the Queen of the Child Welfare and Mothercraft Exhibition in the Central Hall, Westminster. As the Queen entered, she passed along an aisle, where was a "guard of honour" of about a hundred mothers from the different infant centres; and each mother, instead of "presenting arms," "presented her baby." The exhibition contained many items of interest, and among them may be mentioned a series of models (shown by the Royal Free Hospital) of different departments of the hospital, where much attention is devoted to the ante-natal care of mothers. Another exhibit of importance was shown by the Scottish Maternity and Child Welfare Exhibition; it consisted of a series of photographs demonstrating the need for care of the eyes, ears, nose, and throat in young children. The exhibition is well worth seeing; and it is very desirable that it should be visited by large numbers of mothers of the working classes, but unfortunately the admission fee is one shilling during the afternoon, the only time when working mothers with young children can attend. We hope this oversight has been set right in time. A public meeting of health and social workers was held at the Guildhall, under the presidency of the Lord Mayor, at which Lord Rhondda, late President of the Local Government Board, and Mr. Hayes Fisher, his successor, spoke. The Duchess of Marlborough moved a resolution pledging those present to use their influence to secure improved housing and sanitation, and adequate provision for the care of maternity and infancy in their own districts.

Lt.-Col. R. Markham Carter, [L.N.A. Photo.

F.R.C.S. Eng., L.R.C.P. Lond., I.M.S. (see page 263).

### AN ANNUAL BABY WEEK.

The National Baby Week Council have issued an appeal for funds to cover the expenses in connection with National Baby Week, and they suggest that a fund should be started for permanent constructive work. The appeal is signed by the Lord Mayor, Lord Leverhulme, and Mr. William Hardy, and they urge that "Baby Week" should be an annual institution. There is much to be said for the suggestion, and we hope that the result of "Baby Week" this year will demonstrate both the need for such a movement and the value of the prophylactic measures advised.

### HOSPITAL SUNDAY FUND RESULTS.

Approximately the amount received to date at the Metropolitan Sunday Fund office on account of the collection of June 24 is £30,000. This is very good indeed, and compares most favourably with the state of the collection and donation list at the same time after Hospital Sunday in previous years. The plan of organisation so often advocated by The Hospital and dealt with at length in our issue of June 23, page 217, has answered well, and particularly has it been successful in small and poor parishes. There are quite a number of instances of churches in the East End and other working-class districts where the collection is eight and even ten times as much as in 1916. It is true that "every mickle maks a muckle," but while these smaller collections have been increased and the aggregate sum to be distributed appreciably strengthened, the same proportional increase cannot be claimed in the wealthier parishes and congregations. That is not to say that there has been any falling off; indeed, the collections announced to date prove the contrary. It will be appreciated by ministers of religion that systematic organisation as applied to Hospital Sunday is good for the work of the church and chapel as well as that of the hospitals, for it may be taken for granted that a person takes some care in selecting an almoner and has some respect for the agency chosen as the medium of his contribution. It is probable that, as regards the number of individual contributors, Hospital Sunday of 1917 will prove a record. It certainly would have done had the wealthier parishes been organised, too.

### THE DUMPING OF GERMAN WOUNDED.

THE Manchester Board of Guardians has strongly protested against a proposal of the military authorities to accommodate more sick and wounded German soldiers at Withington Hospital. The board opposes the plan on two grounds. The first is the injustice of using the up-to-date equipment of the annexe for Germans while so many British wounded are accommodated in converted buildings not possessing the same special advantages. The second is that in providing about 1,700 beds on the main side of the building f... German wounded the Guardians are already doing their fair share. Unprejudiced people will feel that the second argument is the stronger of the two. The wounded, from whichever army they come, are entitled to the best treatment available, and it is for the military to provide this impartially for all. On the other hand, it is unreasonable to ask any one Board of Guardians to devote its accommodation wholly to the use of German wounded. Feeling is naturally aroused against such a proposal, and the military will only show that they have lost all touch with public opinion if they do not withdraw their demand in the present case.

### THE AMERICAN DOCTORS AT THE FRONT.

WHILE the number and nature of the American Expeditionary Force are kept secret, it is satisfactory to learn details of the medical contingent which has now gone seriously to work in France. According to Reuter, six big British base hospitals have already been taken over by United States units, and some 2,000 medical officers and men are busy at work in the war zone. Add to this the number of American nurses, and the advance guard of the United States Army is seen to be a formidable indication of the battalions which time will bring to France. A United States Army Hospital unit consists of a major in command, an adjutant, and a quartermaster, both of whom have captain's rank. The professional staff is twenty-four in number, apart from the chaplain. There are 216 men of non-commissioned rank, 64 reserve nurses, drawn from American hospitals, a dietetist, and three secretaries. These figures give a general idea of the *personnel* of the average unit.

### THE YORK WORKPEOPLE'S FUND.

THE wisdom of making every hospital the centre of a series of organisations is being proved at York, where the workpeople's hospital fund continues to grow, slowly but steadily. The result of the city collection and the contribution of the country districts has led to rather more than £600 having been raised. The round figure is being given to the York County Hospital. This is an increase of £40 on the grant of the previous year. The fund, in our opinion, has yet possibilities to be developed, independently of any sporadic efforts such as entertainments or house-to-house collections may provide. The centre of interest in workpeople's funds should be in the works themselves, and as these grow in number and in size the movement should spread. Relations between the men and the hospital must be carefully cultivated. The gospel of personal service must be preached by those who understand and believe in it. Every extension or improvement must be so related to the organisations of which the hospital is the centre as to be a source of pride to them. A workpeople's fund must first be a fund of affection if it is also to prove a fund of mercy.

### FATAL SHAVING-BRUSHES.

THE Local Government Board has issued a report drawn up by Dr. F. J. H. Coutts, who has inquired into the deaths from anthrax derived from the use of infected shaving-brushes. The extent of the danger is certainly limited, but there is a very definite risk, and steps should evidently be taken to minimise it as much as possible. There are two precautions which may be taken, one is to prevent the importation of hair from those districts, such as some parts of China and Siberia, where anthrax is common, and the other is to insist on the complete sterilisation of the hair after it is received. It is much more difficult to deal with brushes which have been manufactured abroad, for sterilisation of a brush already made by heat is generally impracticable. Although there have been a good many cases of infection, fortunately the death-rate has been very small. The danger of infection demands precautions, even though the chance of any individual brush being infected may be very slight indeed.

### THE SHORTAGE OF DOCTORS.

No one who has looked into the matter can doubt that at the present time there is a great shortage of doctors in civil life. Everywhere complaints are heard that it is becoming increasingly difficult to obtain suitable candidates for many civil medical posts, and especially the list of panel practitioners is decreasing in an almost alarming fashion. Appeals have been made to panel patients not to call in their panel doctors unless the illness makes it essential; but it is doubtful whether this expedient will prove of any avail, for the selfish patients will persist in invoking medical help whether really needed or not, and the conscientious will not improbably defer asking for help until the delay has done some definite harm. Another method has been suggested for trial in Birmingham. It is proposed to establish a central surgery in the Ladywood district, and at this central surgery only panel patients will be seen. The total number of panel patients entitled to be seen there amounts to about 8,000. A whole-time medical officer is to be in attendance from 9 to 11 in the morning, and from 6 to 8 in the evening, and he will undertake a limited amount of visiting, seeing all cases to which calls are received up to 1 P.M. He will also be responsible for the working of the surgery. When a message comes at any time and no other doctor is available, he will visit the patient himself. The other medical men attached to the central surgery will visit patients from 1 P.M. to 8.30 A.M., and they will also assist the whole-time practitioners, so far as is necessary. It is not proposed to open the surgery on Wednesday evenings and Sundays. The Birmingham Medical War Committee will be responsible for the working of the scheme. One objection has been brought forward which deserves consideration, and that is that some patients would have to travel a great distance to reach the surgery. The scheme is certainly worth consideration, and we shall be interested to see how it works. It is professedly only a war emergency measure.

### RETIREMENT OF A HOSPITAL SECRETARY.

Mr. Richard Ferrier, who has acted for twenty-five years as honorary secretary to the Yarmouth Hospital, has been compelled to send in his resignation. The cause of this is that nearly all his clerks have joined the Forces, so that he is now compelled to devote all his time to his own affairs. Even the clerk at the hospital who used to help him has been called up, and for the past two months he has been assisted by Sir Francis Vincent, who has now become Mr. Ferrier's successor. In acknowledging his election as a life member, Mr. Ferrier declared that Sir Francis possessed not only the necessary leisure but the business experience required for the work. That the hospital possesses public confidence is seen from the fact that, although in January last a deficit of £1,800 was anticipated unless the income were increased, the actual deficit has been under £2. This satisfactory result is due to an effort resulting in a wider list of annual subscriptions to replace the loss of one large figure, and to some generous donations which it is hoped to maintain. Thus Sir Francis Vincent takes up the work on a rising tide, which is the best possible tribute to the record of service rendered by Mr. Ferrier.

### IS THE PENSIONS COMMITTEE TO SEND DISCHARGED SOLDIERS TO THE WORKHOUSE?

The board of the Blackpool Victoria Hospital, owing to the beds being full, has had to decline to receive a discharged soldier suffering from rheumatism. Mr. D. L. Harbottle, clerk to the War Pensions Local Committee, in asking the hospital to admit the soldier, remarked that if he were not admitted he would have to be sent to the workhouse infirmary at Kirkham. That a War Pensions Committee could countenance such an idea, let alone itself send a discharged soldier to the workhouse, is an outrage, for this is precisely the scandal which it is one of the duties of a War Pensions Committee to prevent. Whatever else it does or does not do, it must not do that. Wherever it sends a man, it must not send him there. That it should pretend to have no alternative if a neighbouring voluntary hospital is full is a grave reflection on the Ministry of Pensions. The matter should be pressed on Mr. Barnes, M.P. The board of the Victoria Hospital has done well to place its sense of scandal in the form of a resolution of protest which has been sent to Mr. Barnes, M.P., Mr. Hodge, M.P., Mr. Pringle, and Lord Derby.

### NO COMPULSORY TRAINING FOR WOUNDED.

At a conference of stall-holders in the exhibition of work done by wounded at Messrs. Sotheby's Galleries, Sir Arthur Boscawen, M.P., Secretary to the Ministry of Pensions, said that Mr. Barnes and his Department were anxious to discover whether "it was expedient or possible" to introduce a system of compulsory training in a trade among wounded soldiers in hospital. Some men, he said, on their discharge had formed habits of idleness in hospital, and it was difficult to provide for them. The conference, presided over by Mr. C. A. M. Barlow, M.P., repudiated the notion of compulsion. Lord Charnwood said that it was found in France that very few men learnt anything under compulsion; young fellows might submit, but fathers of families would be found exceedingly refractory. Mr. Myers, of Roehampton Hospital, declared that it was impossible to apply compulsion to men who had lost their limbs. At the end of the discussion Sir Arthur Boscawen seemed to realise that this proposal had better be dropped, for he admitted that when a man was discharged there could be no question of compulsion. Coming from the Ministry of Pensions the proposal has a suspicious air, and every fair-minded person will be glad that it has been repudiated. Inducement remains, the interest of activity remains, and, strongest of all, the boredom of a long period in hospital.

### DISABLEMENT AND DECENTRALISATION.

A conference of representatives of Pension Committees has been held at Norwich with a view to forming a joint committee to deal with naval and military war pensions in the three counties of Norfolk, Suffolk, and Cambridge. The conference was convened by Mr. E. A. B. Barnard, of the Ministry of Pensions, who is inspector in the Eastern Counties division, and was addressed by Major Mitchell, who had come from London to represent the Ministry of Pensions. He explained that his Department was anxious to decentralise the work of treating and training disabled men, and for this purpose it was proposed to establish twenty committees in different areas, which would co-ordinate the work of the 310 local committees formed throughout the country. The Joint Institutional Committee of the Ministry of Pensions is able to provide institutions for disabled discharged sailors and soldiers suffering from paraplegia, epilepsy, neurasthenia, and advanced tuberculosis. Areas will differ in their requirements. The actual duties of these joint committees have yet to be determined, but, broadly speaking, their function is to supplement the work of the local committees wherever these fail to provide for the needs of the disabled in their locality.

### MEDICAL AID FOR POOR WOMEN.

The London County Council is asking the Borough Councils to consider the desirability of making themselves responsible for the payment of the medical fees in those cases where a midwife has to call in a doctor to a difficult labour case, and the authorities are satisfied that the pregnant woman is unable to pay the fee herself. The London County Council points out that the Local Sanitary Authorities have the power to do this under the Notification of Birth (Extension) Act,

1915. The Local Government Board has already expressed its willingness to make a grant of half the amount of any such fees paid by the local authorities.

### THE HOUSING PROBLEM.

It is possible, as a consequence of a fourth housing deputation recently received by Lord Rhondda, to gather some idea of the ultimate proposals of the Government with regard to housing. The President of the Local Government Board went so far as to say that local authorities would be able to rely upon adequate State aid in housing schemes, and he mentioned that at Whitehall he is now being assisted by a committee of experts and officials in preparing schemes so that when the war is over there shall be no delay in setting to work. When Dr. Ethel Bentham mentioned that in Kensington four-fifths of the infantile mortality could be traced to bad housing, as people with children could often only rent basement rooms, Lord Rhondda expressed disappointment. He said it was his ambition to save a thousand infant lives a week, but if that had to depend upon the solution of the housing problem, his ambition would take long to realise. The housing problem could not be solved in a year. It was a matter of time, but nevertheless it was now generally recognised that good health was consequent upon good housing, and the State and municipal authorities were now fully alive to the urgent necessities of the occasion.

### CHESTER CHILDREN LOSING WEIGHT.

In the Medical Officer's report to the Chester Education Committee it is stated that there has been a very definite decrease in the weight of the boys attending the schools. In 1914 the average weight of the boys at the age of eight was 57 lb., while for the year 1916 the average weight was a little under 52 lb., pointing to a drop of over 5 lb. The weight of the boys at the leaving age also shows a marked drop. The average weight of the girls at the age of eight has fallen from 56 lb. to about 49½ lb., and at the leaving age from 80.4 lb. to 69.3 lb. It is suggested that the change in the character of the food may have had something to do with the diminution in the average weight; but statistics must always be received with the greatest caution, and especially statistics dealing with averages. It is quite possible that there may be some entirely different explanation, but we agree with the chairman of the Chester Education Committee that an endeavour should be made to discover the cause, whatever it may be.

### NOTIFICATION OF MEASLES.

For years after most of the common contagious diseases had been notifiable, measles was not included among them. One reason given was that as the disease was so common the expense of notification would be too great; another was that the disease was so mild that it was really not worth notifying. The answer to this second objection is simple. In every week more children die from measles than from all the other contagious diseases combined; measles is certainly not a disease to be despised. A third reason has more support for it. Measles is markedly contagious from a very early stage, several days before the rash appears, and generally by the time the rash has appeared many other children have been infected. Whatever may have been the real cause of excluding measles from the notification, no one can now doubt that it was right to include it. How much good the notification of measles has done it is more difficult to decide; but Dr. C. Court, the medical officer of the Fylde Rural District, is quite sure that it has done good. In his annual report he mentions that the inclusion of measles has greatly increased the number of notifications, especially as the disease has been very prevalent there; but he is satisfied that it has done much good, that it has not only prevented a large number of deaths, but that it has also prevented many cases of disease of the lungs and of the ears, diseases which so commonly follow as sequelæ of measles. He also believes that notification has made the parents attach more importance to measles, as previously the disease had always been regarded as a trivial complaint. It is almost impossible to obtain statistics on such a subject, at all events at present, but it is satisfactory to obtain the conclusions of one who, like Dr. Court, has ample material for forming an opinion.

### A SOLDIER'S LEGACY.

Mr. F. Neden, secretary of the York County Hospital, has reported an interesting bequest from a soldier, which has been transmitted to him in a document from the War Office. This contained a copy of the will of the late Private Fritz Hudson, of the 1-5th Battalion of the Yorkshire Regiment. The will, which was dated July 29, 1916, and duly signed, is thus expressed: "In the event of my death I give £5 to the County Hospital, York, England, and I give the remaining part of my property to my mother and father, Mr. and Mrs. G. T. Hudson, Green, Thirsk, Yorks, England." Oddly, Mr. Neden was unable to trace the testator's name among the files relating to soldiers treated at the hospital, until he communicated with Mr. Hudson, senior, at the address mentioned in the will. In reply he learnt that Private Hudson was a patient in the hospital for about three weeks during October and November 1915, when he was operated on for hernia. He joined the Army early in 1916, which explains the difficulty in finding his name, and was killed in France on March 5, 1917. He was under twenty-one years of age. The estate is estimated at £9 2s. Private Hudson was a working lad before joining the Army.

### WHAT CAUSES ALCOHOLIC EXCESS?

In order to try to solve the riddle "Why do men drink alcohol?" a Research Foundation has been established at Hartford, Connecticut. Dr. T. D. Crothers, in the New York *Medical Record*, gives some indication of the lines upon which the projected research work is to proceed. He believes that the excessive use of alcohol is preceded by distinct physical and psychical causes and forces, that may be studied like any other natural phenomena. For example, says Dr. Crothers, why is it that large numbers of most excellent men suddenly take spirits to great excess for a few days or weeks, then stop and resume their normal condition of healthy, temperate living? The intervals between the drink-storms are marked by most exemplary conduct, while the drink periods are equally prominent in insane and idiotic acts. "Persons of this class," he continues, "are seen in all circles of society, and very often among the most influential, intellectual brain-workers, who are respected and are men of affairs." There is, he says, no explanation of the causes which impel men to drink continuously, even to death. This tendency begins sometimes at the height of prosperity and achievement, or it may follow disaster. On the surface the causes are elation from success or despair from loss and disappointment, but evidently there are some other factors beyond these which must be present to account for the trouble. . . . What conditions of body and mind furnish the soil, plant the seed, and favour the growth of inebriety and alcoholism? These problems must be studied. What laws of growth, heredity, culture, surroundings, nutrition, and mental impression favour the development of the drink evil are questions to be answered. There are general and special causes; there are physical and psychical forces. What they are and how they act; how they develop and culminate; what laws govern their growth and progress—these are the subjects, says Dr. Crothers, which are to be scientifically studied in this new institute.

### THE PRACTICE OF DICTATION.

"The Effect of Printing on Literature" was the subject of an article in The Hospital of April 7, 1917, page 9. The practice of dictation was mentioned in it as an instance of the fact that literature is the one art for which the practice of no handicraft is necessary, and the writer, in dwelling on the effects of this divorce of literature from manual labour, illustrated them by reference to the literary compositions of a certain person of note. When these were written by hand they were concise and clear; when they were dictated they were turgid. In a vivid and well-written sketch of Henry James' method of work in the current *Fortnightly*, Miss Theodora Bosanquet, his former amanuensis, refers to his practice of dictating his later volumes. "I do not know," she writes, "when Henry James began the practice of dictating his work, but I think it must have been in '95 or '96." Its effect on his style, she says, he fully recognised; "to a certain extent he even deplored it. 'I tend,' he once said, 'to be too diffuse when I am dictating.' . . . He considered [we learn] that the increased facility more than made up for any loss of concision. There were, however, certain kinds of composition that he found himself obliged to work at with his pen. Plays and short stories, if they were to remain within the bounds of possible publication in a magazine, he usually wrote by hand, knowing that the manual labour of writing would be his best aid to the desired brevity." This is a pregnant illustration of the force of our contributor's contentions. Henry James' naturally decussated style deteriorated through his practice of dictation into a mere reticulation of parentheses, and if such a practised writer as he could not afford to neglect the homely handicraft of penmanship, the ordinary man of affairs, whose aim should be brevity, may learn from James' experience the potent effect of handwriting on the art, and even the decency, of letters.

### POOR-LAW INSTITUTIONAL TREATMENT AND THE NATIONAL INSURANCE ACT.

The Local Government Board have transmitted to the Lambeth Board of Guardians a letter received by them from the National Insurance Commission (England) on the subject of the form of the medical certificate issued to insured persons in the infirmary to enable them to claim sickness benefit. The certificate issued from the infirmary is to the effect that the person who is the subject of it is an inmate of the institution, and the Commissioners state that a claim made by an insured person for sickness benefit under the National Insurance Act has been refused by an approved society owing to the omission from the certificate of any statement of the disease or disablement from which the patient is suffering, and in respect of which he is receiving treatment in the institution. The Local Government Board, in forwarding the letter, state that they consider that Article 59 (10) of the Poor-Law Institutions Order, 1913, requires the medical officer to give to an inmate of the institution on his request a certificate in which the nature of his illness or other cause of the medical officer's attendance on him is stated.

### DISAGREEMENT OF THE OFFICERS WITH THE BOARD.

The Clerk to the Guardians informed them that the Local Government Board was in error in quoting the Poor-Law Institutions Order in this connection, for in the preamble to the Order it is provided that "nothing contained in this Order shall apply to a separate institution which is provided wholly for the reception and maintenance of persons suffering from disease of mind or body." It was obvious, therefore, that the Article to which they referred did not impose any obligations on the medical officer of the infirmary in this respect. The Clerk added that from a careful perusal of the Special Order of the Local Government Board issued to the infirmary in which are defined the duties of the medical officer, he could not find that the medical officer was required to issue a medical certificate to a patient. The Guardians have agreed, before coming to any decision on the matter, to place these points before the Local Government Board, and to ask whether there was any Order which they considered required the medical officer of the infirmary to act in the manner suggested.

### THE FIRE AT THE FIRST LONDON.

THANKS to the activity of the staff, which began to work a hydrant before the arrival of the fire brigade, the outbreak which started in the general stores of the 1st London General Hospital, Akerman Road, Brixton, was confined to these buildings, and did not spread to the main block. The canteen and the stock in the yard were, however, badly damaged, while the building in which the stores were kept was virtually destroyed. The fire is said to have been caused by the dropping of a light, and the wards had a narrow escape in spite of the arrival of engines from four stations.

### THE LIGHTER SIDE.

THE 1st Southern General Hospital, Birmingham, has entered on a new phase with the creation of the post of A.D.M.S. of the Birmingham area. Colonel Marsh, its administrator, has been appointed to this, which is the latest step in the expansion of the hospital which has taken place during the past two years. All the medical services in Warwickshire and Worcestershire are included in the area under the control of the new office, which may bring more recruits to the contributors of the *Southern Cross*, a well-filled "gazette" now approaching the end of its second volume. The June number was mostly filled with short sketches, including a skit on a wealthy and pompous visitor, and some humorous lines chaffing a nurse for wearing silk stockings. If people can be induced to touch lightly on such themes with sufficient skill to convey their feelings to others, the lighter side of hospital life can be presented; and since trifles of the kind do more than anything else to pass the time pleasantly, they deserve to have a larger place than they usually receive in these "gazettes." The trouble is not only to get them well described, but to find editors unfettered enough to pass them.

### BRIGHTON AND THE BABIES.

IN connection with Brighton and Baby Week there was much interest at what was really the inaugural ceremony, in the visit of Sir Arthur Newsholme, for not only is he the chief medical officer attached to the Local Government Board, but he was for nearly twenty years medical officer of health for Brighton. There are lasting memorials of Sir Arthur Newsholme's work at Brighton—the sanatorium on the Race Hill, the abattoir, and the district which once was a great slum area, are only a few that come to one's mind. And, coupled with Baby Week of 1917, it will not be surprising to find another memorial of Dr. Newsholme's work at Brighton—namely, that the Corporation takes the earliest opportunity of removing the reproach that last year Brighton spent only £226, including the salaries of the health visitors, in direct work towards the preserving of child-life. "In order to save life," Sir Arthur said, "you must spend money; and it is a good investment." In Brighton a halfpenny rate produces £1,800, and surely this even would be a small enough sum to spend annually in the work of reducing infantile mortality. Perhaps, after Baby Week, with all its meetings, demonstrations, etc., there will be found a considerable decline in the opposition that has heretofore existed to voting money for this purpose, despite the figures and appeals which Dr. Duncan Forbes, the present medical officer of health, has presented to the Corporation.

### THE NEW TROUBLE.

SCABIES is the latest disease to embarrass local authorities. Reports from all quarters indicate an unusual prevalence of this complaint. In West Ham the Board of Guardians and the sanitary authorities have arranged for a conference to discuss the question of the treatment of scabies. The L.C.C. reports that there are many cases in Deptford, and is arranging to open a cleansing station for the bathing of children. After all, the treatment of scabies is very simple, and the main risk in the treatment is the chance of setting up sulphur dermatitis, which is mistaken for a continuance of the disease.

### WAR BREAD.

THE question of the effect on the public of the changes which have been made in the composition of our daily bread is by no means easy to answer, and yet it is a matter of no little importance, not only to those in good health, but even more to those who are ill, and therefore the managers of hospitals and similar institutions have had to consider carefully what effect the change in the composition of the bread may have on those within their walls. There is no doubt that many complaints have been made against war bread, and that accusations of its having been the cause of many symptoms have been widely spread, but it is more difficult to prove that these accusations have a real basis. The matter would be less difficult if the composition of the war bread were more uniform, but in truth the bread that we can now obtain by purchase may vary greatly. All that the regulations insist is that the bread should not contain more than a certain percentage of wheaten flour; what the rest of the loaf may consist of is left to the fancy of the miller or baker. Those who have given much attention to the matter are inclined to believe that in some cases, at least, there is a very real basis for the accusation; but even if this be allowed, it is still more difficult to say what is the particular constituent which is generally responsible for the injurious effect. There is some evidence that the most obnoxious addition is bean meal, while barley meal and ground rice may be added with little impairment of the taste and no real reduction in the nutritive value of the loaf, and moreover there does not seem to be any evidence that these flours give rise to any harmful symptoms. We think that it would be well that some suitable authority should inquire into this question, for, other things being equal, it is clearly desirable that attention should be paid to the point of digestibility of the war bread, even though it is quite certain that some admixture with wheat flour must be made.

## Figures and Tables

**Figure f1:**